# Vitamin E as an Antiosteoporotic Agent via Receptor Activator of Nuclear Factor Kappa-B Ligand Signaling Disruption: Current Evidence and Other Potential Research Areas

**DOI:** 10.1155/2012/747020

**Published:** 2012-08-02

**Authors:** Kok-Yong Chin, Soelaiman Ima-Nirwana

**Affiliations:** Department of Pharmacology, Faculty of Medicine, Universiti Kebangsaan Malaysia, Jalan Raja Muda Abdul Aziz, 50300 Kuala Lumpur, Malaysia

## Abstract

Osteoporosis is a growing healthcare burden that affects the quality of life in the aging population. Vitamin E is a potential prophylactic agent that can impede the progression of osteoporosis. Various *in vivo* studies demonstrated the antiosteoporotic potential of vitamin E, but evidence on its molecular mechanism of action is limited. A few *in vitro* studies showed that various forms of vitamin E can affect the receptor activator of nuclear factor kappa-B ligand (RANKL) signaling and their molecular targets, thus preventing the formation of osteoclasts in the early stage of osteoclastogenesis. Various studies have also shown that the effects of the different isoforms of vitamin E differ. The effects of single isoforms and combinations of isoforms on bone metabolism are also different. Vitamin E may affect bone metabolism by disruption of free radical-mediated RANKL signaling, by its oestrogen-like effects, by its effects on the molecular mechanism of bone formation, by the anti-inflammatory effects of its long-chain metabolites on bone cells, and by the inhibition of 3-hydroxyl-3-methyglutaryl coenzyme A (HMG-CoA). In conclusion, the vitamin E isoforms have enormous potential to be used as prophylactic and therapeutic agents in preventing osteoporosis, but further studies should be conducted to elucidate their mechanisms of action.

## 1. Introduction

Osteoporosis is a metabolic disease of the skeletal system, characterized by low bone mass and deterioration of the microarchitecture of bone, leading to fragility and subsequent fractures [1]. According to an estimate done in the year 2000, nine million osteoporotic fractures occurred globally among men and women aged 50 years and above, in which 1.6 million were at the hip, 1.7 million at the forearm, and 1.4 million at the vertebrae. Most of the osteoporotic fractures occurred in Europe (34.8%), followed by the Western Pacific region, inclusive of East Asia, Australia and New Zealand (28.6%) and Southeast Asia (17.4%) [2]. With the global shift of distribution of death from younger to older ages and from communicable to noncommunicable causes [3], osteoporosis presents a growing disease burden especially in developing countries [4].

Both men and women suffer from osteoporosis, but the prevalence of osteoporosis, as indicated by osteoporotic fractures, is lower in males than in their female counterpart. The ratio of female to male osteoporotic fractures is 1.6 (61% of fractures occur in women) [2]. The major cause of osteoporosis in women is due to the presence of a phase of accelerated bone loss during menopause, which accounts for 20–30% loss of cancellous bone and for 5–10% loss of cortical bone. Despite the absence of a similar rapid bone loss in women, aging men do experience a gradual phase of bone loss which contributes to 20–25% in both cortical and cancellous bone [5]. This decline has also been demonstrated in our previous study using the calcaneal quantitative ultrasound technique in Malaysian men [6]. While bone loss in women is caused by the decline of sex steroids during menopause, bone loss in men is contributed by the decrease in bioavailability of testosterone due to the increase in sex hormone-binding globulin level with aging [5]. The assessment of age-related changes of microarchitecture in humans is made possible due to the advancement of high-resolution peripheral quantitative computed tomography (HR-pQCT). A study by Khosla et al. reported that a cross-sectional reduction in bone volume (BV/TV) at the radius was observed in men and women. The decrease in trabecular number (Tb.N) and increase in trabecular separation (Tb.S) were more prominent in women while the reduction in trabecular thickness (Tb.Th) was more evident in men [7].

Apart from age-related hypogonadism and menopause, osteoporosis also develops as a result of the following causes (Table 1).

According to an estimate by Burge et al., the economic cost of osteoporosis (inclusive of long-term care cost) in the United States was more than 16.9 billion dollars. This cost was projected to increase to 25.3 billion dollars (an increment of 48% compared to the year 2005) by the year of 2025 due to the increase in the age of the population and bone fracture incidence [8]. In the European Union, the cost of osteoporotic fractures was € 30.7 billion in 2010 and was expected to rise to € 38.5 billion in 2025. Apart from the direct hospitalization cost and long-term care cost, the burden of osteoporosis can be measured in years of life lost due to a fracture and the disability (disability-adjusted life years/DALYs). The total DALYs lost due to osteoporotic fractures globally were estimated to be 5.8 million, which were around 0.83% of the global burden of noncommunicable diseases [9]. Unfortunately, data for health economic burden of osteoporosis in Malaysia is currently not available.

## 2. RANKL Signaling and Osteoclastogenesis

Bone is constantly under deconstruction (resorption) and reconstruction (formation) in a process called bone remodeling. Osteoporosis occurs when the rate of bone resorption exceeds the rate of bone formation [10]. Macrophage colony-stimulating factor (M-CSF) and receptor activator of NF-*κ*B ligand (RANKL) are cytokines secreted by osteoblast and stromal cells which are important for proliferation, differentiation, and maturation of osteoclasts [11]. M-CSF binds to colony-stimulating factor-1 receptor (c-Fms) on the osteoclast precursors and primes them to differentiate in the presence of RANKL by inducing RANK expression [12]. Humans with mutation in the gene encoding RANKL will suffer from osteopetrosis, and their bone specimens lack osteoclasts [13]. Binding of RANKL to RANK induces several signaling pathways eventually leading to the activation of genes required for osteoclast differentiation and activation. Several transcription factors, such as nuclear factor kappa-B (NF-*κ*B) (p50 and p52), nuclear factor of activated T cells, cytoplasmic, calcineurin-dependent 1 (NFATc1), and FBJ osteosarcoma oncogene (c-Fos) function downstream of RANKL signaling and the pathways are mediated by protein kinases, such as sarcoma oncogene (Src), c-Jun N-terminus kinases (JNK), p38 mitogen-activated protein kinase, and phosphoinositide 3-kinase (PI3K). NF-*κ*B has been shown to control RANKL-induced osteoclast differentiation, leading to activation of c-Fos prior to NFATc1 [14]. NFATc1 is also called the master switch of terminal osteoclast differentiation because without NFATc1 stem cells cannot differentiate into osteoclasts even in the presence of RANKL, and this can be reversed by exogenous NFATc1 [15].

Osteoprotegerin (OPG) is a protein that exerts protective effects on bone by binding to RANKL, thus interrupting osteoblast-osteoclast progenitor signaling and subsequently preventing osteoclast activation and differentiation [18]. Proinflammatory cytokines, such as tumor necrosis factor alpha (TNF*α*), interleukin 1*β* (IL1*β*), and interleukin 6 (IL6), are known to increase both RANKL and OPG expression, but the dominant outcome is a net increase in RANKL activity. They also act synergistically with RANKL in regulating osteoclastogenesis [19]. Maximal expression of RANKL under the influence of these cytokines may require p38 mitogen-activating protein kinase (MAPK) pathway [20]. Prostaglandin E_2_ (PGE_2_) has also been implicated in the upregulation of RANKL, as shown in increased RANKL mRNA and cell-surface RANKL protein in mouse pre-B cell line after treatment, which is probably caused by TNF*α*/IL1 induction of cyclooxygenase (COX) activity [21]. PGE_2_ downregulation of OPG is also implicated in lipopolysaccharide-induced osteoclastogenesis but in the same experiment, upregulation of RANKL is independent of PGE_2_ [22]. Other factors that can influence RANKL/OPG ratio include parathyroid hormone (PTH), vitamin D3, transforming growth factor *β* (TGF*β*), bone morphogenic protein (BMP) 2, glucocorticoids, 17*β*-estradiol, and insulin-like growth factor-1 (IGF-1) [11].

The reactive oxygen species (ROS) play a critical role in RANKL signaling. Hydrogen peroxide (H_2_O_2_), a ROS originating from the dismutation of superoxide, has been shown to induce differentiation of osteoclast precursor cells [23]. Thioredoxin-1 (Trx-1) also increases the capability of precursor cells to differentiate into osteoclasts and transfection with glutathione peroxidase-1 (Gpx-1), and peroxiredoxin (Prx-1) abolishes osteoclast formation. Trx-1 also augments RANKL-induced activator protein 1 (AP-1), NF*κ*B, and NFAT gene expression in preosteoclasts [24]. Pretreatment with N-acetyl-L-cysteine (NAC) and GSH have also been shown to reduce RANKL-induced Akt activation, and pretreatment with NAC alone inhibits degradation of I*κ*B*α* (which inhibits NF-*κ*b) and RANKL-induced MAPKs (extracellular signal-regulated kinase/ERK, JNK, and p38) activation. Actin ring formation and bone-resorbing activity of osteoclasts are also inhibited by NAC [25]. Further study revealed that blocking of nicotinamide adenine dinucleotide phosphate (NADPH) oxidase (Nos) will impair RANKL signaling in bone morphogenic macrophage (BMM) cells by blocking the intracellular ROS production, activation of ERK, JNK, and p38, and ultimately preventing osteoclast differentiation [26].

From the information above, it is clear that an ideal agent to prevent osteoporosis should be able to interrupt RANKL and its downstream signaling pathway, by means of suppressing proinflammatory cytokine-induced RANKL expression or downregulation of OPG, or by disrupting ROS-mediated RANKL signaling.

## 3. Therapy for Osteoporosis

The current therapy for osteoporosis can be classified into antiresorptive therapy (example: bisphosphonates and estrogen) and anabolic therapy (e.g., teriparatide). Antiresorptive drugs like nitrogen-containing bisphosphonates inhibit farnesyl diphosphate synthase, thus reducing osteoclast activity. They also promote osteoclast apoptosis by activation of proapoptotic caspases [28]. There are some concerns that bisphosphonates may increase the risk of osteonecrosis of the jaw [27]. Estrogen exhibits both antiresorptive and anabolic properties because it acts on the osteoclastic and osteoblastic compartments. Estrogen reduces the responsiveness of osteoclast progenitor cells to RANKL and subsequently hinders their differentiation. It also improves osteoblast proliferation, differentiation, function, and lifespan [28]. However, the side effects of long-term estrogen use such as increased risk of breast cancer and cardiovascular disease may out weight its benefits [29]. Teriparatide functions as an anabolic agent by acting directly on osteoblastic lineage cells and indirectly through regulation of skeletal growth factors like insulin growth factor 1. However, teriparatide is very expensive, so it may not be affordable for every patient. It is also contraindicated in patients with hypercalcemia, Paget's disease, skeletal metastases, or skeletal malignant conditions and young patients [30].

Although newer pharmacological agents in the treatment of osteoporosis with fewer side effects have been introduced, the cost and accessibility of the treatment may present a barrier to the patients, especially in developing countries. The quest to find new agents which can concurrently prevent and reverse the progression of osteoporosis, reduce the disease burden of the healthcare system and the patients, and improve the quality of life in the aging population is imperative. The recent discovery of the anti-osteoporotic properties of plant oil-derived vitamin E may offer a solution to the problem. This paper is aimed at discussing the molecular mechanisms leading to osteoclastogenesis and subsequently osteoporosis, and how vitamin E could affect these pathways. All the current evidence pertaining to the antiosteoporotic properties of vitamin E and some suggestions for future studies are also presented.

## 4. Vitamin E and Its Bone Protective Properties

**Figure 1:**
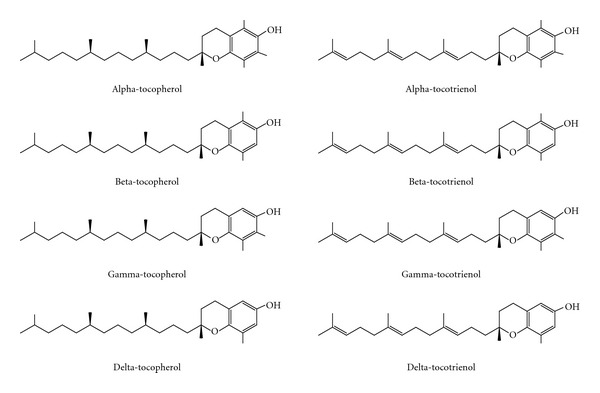
Chemical structures of tocopherols and tocotrienols.

Vitamin E is a fat-soluble vitamin first isolated in green leafy vegetables. It consists of 2 major isoforms: tocopherols and tocotrienols, each with four distinct analogues (alpha, beta, gamma, and delta) due to differences in the location of methyl groups on the chromanol ring. Tocopherols are saturated forms of vitamin E, and tocotrienols are the unsaturated forms, distinguishable by the three double bonds in the tails of tocotrienols. Tocopherols and tocotrienols are commonly found in edible plant oils, such as rice bran, coconut, palm, and annatto oil in varying proportions [31]. The structures of the various vitamin E isomers are depicted in Figure 1.

The importance of vitamin E on bone metabolism was depicted when rats fed with vitamin E-deficient diet had lower calcium content in the left femur and L5 vertebra compared to control rats, and this was prevented by supplementing the rats with palm vitamin E containing alpha-tocopherol (ATF), alpha-tocotrienol (ATT), gamma-tocotrienol (GTT), and delta-tocotrienol (DTT) for eight months. Supplementation with ATF alone failed to demonstrate similar effects [32]. Further study indicated that vitamin E-deficient diet caused hypocalcaemia and subsequently hyperparathyroidism and decreased calcium content in the fourth lumbar vertebra in rats [33]. Normal male rats supplemented with various vitamin E isomers also displayed better structural, static, and dynamic bone histomorphometry, as well as better biomechanical characteristics compared to control rats, with GTT showing the best effect compared to ATT and DTT [34, 35]. Studies in male mice showed that high-dose ATF supplementation (500 mg/kg) could increase mRNA transcripts of osteocalcin and insulin-like growth factor in young and old mice. In addition to that, improvement in biomechanical strength was best demonstrated in old mice rather than in young mice. However, there was no improvement in the bone mineral density of the mice in that study [36]. Supplementation of palm-tocotrienol containing ATT, GTT, DTT, and ATF at 100 mg/kg for four months was shown to improve redox status in bone by decreasing thiobarbituric acid-reactive substance (TBARS) and increasing glutathione peroxidase activity in the femur of rats. This effect was not seen in rats supplemented with ATF alone [37].

Vitamin E also showed beneficial effects on various animal models of osteoporosis. Studies showed that tocotrienol-enriched fraction (TEF, containing ATT, GTT, and DTT), GTT alone and ATF alone exerted beneficial effects on nicotine-induced osteoporosis in male rats by improving bone structural, cellular, and dynamic histomorphometric measurements, and the effects of GTT alone were more superior than TEF and ATF alone [38]. This effect might be contributed by the fact that nicotine-induced increase in bone-resorbing cytokines (IL1 and IL6) was abrogated by TEF and GTT supplementation, and this was indicated by lower urinary pyridinoline (bone resorption marker) and higher serum osteocalcin (bone formation marker) levels in the supplemented group as compared to the control group. ATF was also found to suppress the elevation of IL1 but not IL6, and it could not prevent the rise in pyridinoline. This might be the reason why GTT exerted better anti-osteoporotic effects than ATF [39]. Tocotrienol mixtures containing ATT, GTT, and DTT protected rodent bone from ferric-nitrilotriacetate- (FeNTA-) induced osteoporosis as indicated by the lower level of IL6 and deoxypyridinoline cross-links (DPD) and better histomorphometric parameters in the tocotrienol-treated groups compared to the control group. In the same experiment, it was also found that the tocotrienol mixture was better than ATF alone in preventing free radical-induced osteoporosis [40]. A study by Ima-Nirwana and Suhaniza showed that side effects of long-term glucocorticoid usage were prevented by GTT. When adrenalectomized rats given excessive glucocorticoids were supplemented with GTT, their calcium content in the fourth lumbar vertebrae was higher than the unsupplemented group. This effect was not shown in rats supplemented with ATF [41]. In another study, supplementation of palm vitamin E mixture containing ATF, ATT, GTT, and DTT to rats also prevented dexamethasone-induced osteoporosis by preventing BMD (whole body) and bone calcium loss at femur, but no comparison between mixtures and single isomers was conducted [42]. High-dose of ATF (500 IU/kg diet) could also prevent osteoporosis induced by hindlimb unloading in male rats, shown by improved bone histomorphometry compared to the control group. These effects could be contributed by suppression of COX-2 enzyme expression and a modest increase in bone formation by high-dose ATF [43]. In an orchidectomized rat model, supplementation with palm olein and palm vitamin E containing ATF, ATT, GTT and DTT prevented BMD loss at various sites and bone calcium loss at L5 [44]. In a recent study, tocotrienol-rich fraction containing ATF, ATT, GTT, and DTT was shown to be more effective than oestrogen-replacement therapy in preventing osteoporosis in ovariectomized rats, indicated by improvement in bone histomorphometric parameters in the supplemented female rats [45]. A study by Norazlina et al. showed that supplementation with palm tocotrienols containing ATF, ATT, GTT, and DTT could improve femoral bone calcium levels in both intact and ovariectomized female rats while supplementation with ATF at the same dose could only increase bone calcium content in intact female rats [46]. Nazrun et al. later showed that palm tocotrienol mixtures (containing ATT, GTT, and DTT) could improve bone mechanical strength of ovariectomized female rats, and this could be contributed by reduced lipid peroxidation and induction of superoxide dismutase and glutathione peroxidase in the bone of the supplemented group. The researchers also found that the effects of supplementation with tocotrienol mixture were better than with ATF alone [47].

Overall, animal studies clearly indicate that vitamin E has anabolic effects on the skeletal system and may preserve bone health. This may be brought about by vitamin E-mediated suppression of bone-resorptive cytokines and elevation of antioxidant enzyme activity in bone, which ultimately disrupt RANKL signalling. However, the exact mechanism in which vitamin E, especially tocotrienols, prevents osteoporosis is still not completely understood and needs further studies. The differences in the anti-osteoporotic effects among different vitamin E isomers, and among different vitamin E mixtures are obvious, but the reason was not fully explored. There is also some disagreement regarding the anti-osteoporotic potential of ATF, in which some studies showed beneficial effects [43], while the others found none [41] or lesser effects [38, 40] compared to tocotrienols.

## 5. The Effects of Vitamin E on RANKL Signalling

In a bone marrow cell-osteoblast coculture, Trolox, a hydrophilic derivative of ATF with a carboxylic group instead of a phytyl chain, was found to suppress IL1, COX-2 enzymatic activity and subsequently PGE_2_ production without affecting the expression of COX-2, phospholipase A2, and membrane-associated PGE synthase-1. IL1-induced RANKL expression was also suppressed by Trolox. It prevented osteoclast formation in the early stage (first two days in the coculture) but had no effects on mature osteoclasts. In addition, Trolox could abolish RANKL-induced c-Fos and NFAT2 protein expression without marked change in c-Fos mRNA expression. However, it did not have any effects on IL1-activated signalling pathways involving ERK, PI3K/AKT, and p38 [48].

The antiresorptive potential of ATT was demonstrated in a bone marrow macrophage- (BMM-) osteoblast coculture, in which osteoclast formation was inhibited by suppressing RANKL expression without affecting OPG expression. ATF was found to have no effects on the expression of both proteins. Treatment of BMM cells with ATT inhibited osteoclast formation, but it was only effective when added in the early stage of osteoclastogenesis (first two days in culture). ATT was discovered to suppress RANKL-induced mRNA and protein expression of c-Fos and NFATc1, acute activation of ERK (but not on JNK and p38), NF-*κ*B activation, and pit formation (indicative of osteoclast resorptive activity) on dentin-coated plate by mature osteoclasts [49].

In contrast to the studies previously mentioned, a recent study indicated that megadose of ATF may promote osteoclastogenesis and affect bone health adversely. When normal mice and rats were supplemented with 600 mg/kg ATF (ten times higher than the effective dose used by other researchers) for eight weeks, a 20% decrease in bone mass, with concomitant increase in bone resorption and osteoclast size compared to mice/rats fed with normal diet was observed. This effect was not seen with mice supplemented with delta-tocopherol. Mice deficient in alpha-tocopherol transfer protein (TTPA), which selectively transfer alpha-tocopherol into lipoproteins, were also found to have higher bone mass compared to wild type mice and this could be reversed by alpha-tocopherol supplementation. Further *in vitro* investigation revealed that p38 pathway might be involved in osteoclast fusion and only ATF promoted osteoclast fusion, but other vitamin E isomers including ATT did not exhibit such effect. Other antioxidants, including ascorbic acid, did not also stimulate osteoclast fusion, indicating this effect of ATF was not due to its antioxidant properties [50].

**Figure 2:**
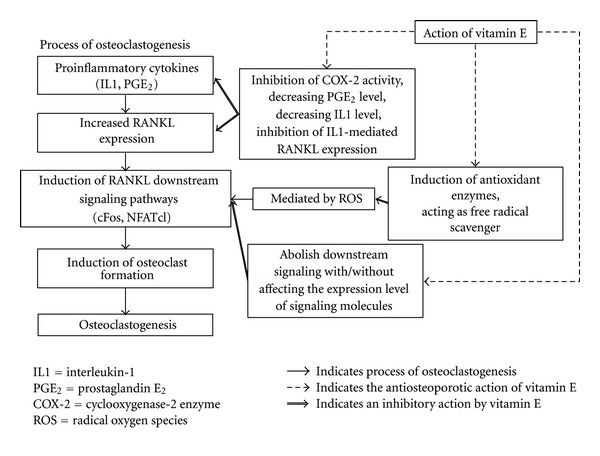
Mechanism of action of vitamin E in preventing osteoclastogenesis. Vitamin E affects osteoclastogenesis via three distinct mechanisms. Firstly, it inhibits COX-2 activity and subsequently PGE^2^ level. It also decreases IL-1 level and thus preventing IL-1-mediated RANKL expression. Secondly, vitamin E induces upregulation in antioxidant enzymes in bone and acts as a free radical scavenger itself, thus abolishing ROS-mediated RANKL signaling. Lastly, vitamin E also abrogates downstream signaling pathways leading to osteoclastogenesis even with or without affecting the expression level of signaling molecules.

Overall, the *in vitro* experiments describing the mechanism of action of vitamin E on regulating osteoclastogenesis are very limited. The studies presented indicated that Trolox and ATT can inhibit RANKL signaling by suppressing RANKL expression although the mechanism of action downstream may vary slightly. Not all the vitamin E isomers were tested in the same experiments, hence, their effects cannot be directly compared. The adverse effects of high-dose ATF are alarming. This may be contributed by the fact that increasing ATF levels, in the presence of oxidative stress and without a balance network of antioxidants, may result in an increase of ATF free radicals which cannot be reduced by other coantioxidants [51]. In addition, an *in vitro* study by Nizar et al. found that GTT at low dose (1 *μ*M) was protective against free-radical damage on osteoblasts, but concentration beyond 50 *μ*M was toxic to osteoblasts. ATF was not protective to osteoblast for free-radical damage at all doses used [52]. The overall mechanism of action of vitamin E is summarized in Figure 2.

## 6. Potential Areas of Interest in Vitamin E-Mediated Bone Remodeling

**Figure 3:**
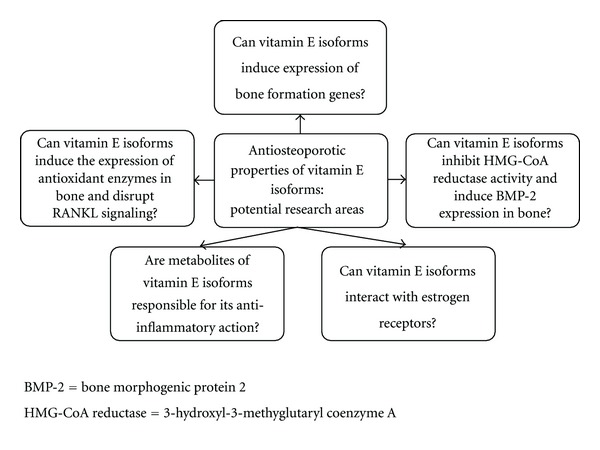
Potential research areas for the anti-osteoporotic properties of vitamin E isoforms.

The research on the molecular actions of the vitamin E isoforms has just begun, and there are plenty of uncharted territories left for future exploration (as highlighted in Figure 3). Vitamin E, especially the tocotrienol fraction, is known to induce expression of antioxidant enzymes and quench ROS in tissue. Rats supplemented with vitamin E showed elevated concentrations of liver superoxide dismutase, glutathione peroxidase, and catalase compared to the control group [53]. We have also shown that palm-tocotrienol mixture containing ATT, GTT, and DTT increased glutathione peroxidase level in femoral bones of supplemented rats [37]. Besides that, a study by Norazlina et al. indicated that ATF and tocotrienol mixtures (content not specified) can prevent bone calcium loss due to nicotine without affecting serum RANKL to OPG ratio significantly [54]. Whether this induction of antioxidant enzymes transpires in bone in the presence of vitamin E, and whether it is responsible for the disruption in RANKL signaling independent of the expression of RANKL need validation via *in vitro* studies. 

A recent study also revealed that tocotrienols (GTT, DTT, and tocotrienol-rich fraction) display high affinity for estrogen receptor beta (ER*β*) and interact with it by increasing its translocation into the nucleus and activating estrogen-responsive genes in breast cancer cells [55]. Since previous studies found that gamma-tocotrienol showed anabolic effect on the skeleton of normal male and female rats [34, 35], it is reasonable to speculate whether it has similar estrogenic effects on bone. Apart from that, although some effects of vitamin E on osteoclastogenesis have been studied, information pertaining to its effects on the molecular regulation of bone formation, such as on bone formation genes in osteoblast is lacking and should be studied.

 There are plenty of evidence that vitamin E can suppress the expression of proinflammatory cytokines which promote osteoclastogenesis, as reviewed by Nazrun et al. [56]. A recent finding suggested an alternative to the mechanism of the anti-inflammatory action of vitamin E. A study on inhibition of the cyclooxygenases (COX) by vitamin E revealed that long-chain carboxychromanols (metabolites of vitamin E), rather than vitamin E itself was the potent inhibitors of prostaglandin E_2_ synthesis in human lung epithelial A549 cells. The inhibitory effect of vitamin E was lost when its metabolism in cells is inactivated and the metabolites are not formed. Computer simulation done on enzyme kinetics data showed that metabolites of vitamin E competed with arachidonic acid to bind with COX, and the 13′-COOH fraction of carboxychromanol was more potent than 9′-COOH fraction in enzyme inhibition [57]. Whether these vitamin E metabolites were beneficial in suppressing bone damage due to inflammation and RANKL signaling would be an interesting field of investigation.

It is known that statins, a class of lipid lowering drugs which act as 3-hydroxyl-3-methyglutaryl coenzyme A (HMG-CoA) reductase inhibitor can reduce fracture risk in the elderly population [58–60]. It was shown that statins enhanced BMP-2 mRNA expression in bone cells and induced bone formation [61], and the addition of mevalonate (a metabolite downstream of HMG-CoA reductase) could abolish the activation of BMP-2 promoter [62]. These effects were not seen in hydrophilic statins [62]. Tocotrienols such as GTT was found to cause posttranscriptional HMG-CoA expression in hepatocyte culture [63]. *In vivo* studies also found that tocotrienol mixtures (containing ATT, GTT and DTT) derived from palm oil fatty acid distillate (PFAD) can inhibit HMG-CoA reductase activity [64]. However, Ng and Khor. showed that the inhibitory activity of tocotrienols could be decreased in the presence of ATF [65]. Thus, part of the anti-osteoporotic effects of tocotrienols could be attributed by its inhibitory action on HMG-CoA reductase enzyme similar to the statins, but direct evidence is needed to elucidate the relationship between them.

## 7. Conclusion

Vitamin E, either together or separated into its various isomers, has enormous potential to be developed as an anti-osteoporotic agent. Animal studies have provided positive outcomes which include improvements in the majority of the histomorphometric parameters as well as biomechanical strength. However, its mechanisms of action at the molecular level need to be elucidated. The current evidence indicates that Trolox and ATT can disrupt RANKL and its downstream signaling thus preventing the formation of osteoclasts in early osteoclastogenesis. Other indirect evidence provided by application of various isoforms of vitamin E in cell lines other than bone cells also suggests vast uncharted research areas for the study of the anti-osteoporotic properties of vitamin E. Apart from that, current studies showed that different isoforms of vitamin E have different effects in bone metabolism. Thus, it is important to study each isoform on its own, as well as in combination. The properties of these isoforms may differ when given individually or in combination since there may be a previously unrecognized interaction among them. Furthermore, due to their chemical structure, the properties of the tocotrienols may differ vastly from the tocopherols and even be opposing in certain situations. Thus, there is a real need to look at the tocopherols and tocotrienols as different chemical entities and not as a single entity under the general name of vitamin E.

## Figures and Tables

**Table 1:** Secondary factors of osteoporosis.

Drug
Aluminium
Anticonvulsants (phenobarbital, phenytoin)
Antidepressants
Aromatase Inhibitors
Cytotoxic drugs
Glitazones (antidiabetic agent)
Glucocorticosteroids and adrenocorticotropin
Gonadotropin-releasing hormone agonists
Heparin
Immunosuppressants
Lithium
Loop diuretics
Proton-pump inhibitor
Thyroxine
Endocrine Disorders
Hyperparathyroidism
Hyperthyroidism
Immobilization
Inflammation
Rheumatoid arthritis
Inflammatory bowel disease
Ankylosing spondylitis
Other factors
Alcoholism
Coeliac disease
Gastrectomy
Prostate cancer therapy

(Source: National Osteoporosis Foundation 2003 [16], Kok and Sambrook 2009 [17]).
